# Fabrication and Arc Erosion Behavior of Ag-SnO_2_-ZnO Electrical Contact Materials

**DOI:** 10.3390/ma16103618

**Published:** 2023-05-09

**Authors:** Danny Guzmán, Felipe González, Diego Muranda, Claudio Aguilar, Alexis Guzmán, Álvaro Soliz, Lorena Lozada, Iñigo Iturriza, Felipe Castro

**Affiliations:** 1 Departamento de Ingeniería en Metalurgia, Universidad de Atacama, Av. Copayapu 485, Copiapó 1530000, Chile; felipe.gonzalez.14@alumnos.uda.cl (F.G.); alexis.guzman@uda.cl (A.G.);; 2 Departamento de Ingeniería Metalúrgica y de Materiales, Universidad Técnica Federico Santa María, Av. España 1680, Valparaíso 2340000, Chile; 3 Centro de Estudios e Investigaciones Técnicas de Gipuzkoa (CEIT-IK4), 20018 San Sebastián, Spain; 4 Departamento de Ingeniería Metalúrgica, Universidad de Santiago de Chile, Av. Lib. B. O’Higgins 3363, Santiago 9160000, Chile

**Keywords:** electrical contact materials, hot pressing, Ag-SnO_2_-ZnO composites

## Abstract

This study investigated the synthesis of Ag-SnO_2_-ZnO by powder metallurgy methods and their subsequent electrical contact behavior. The pieces of Ag-SnO_2_-ZnO were prepared by ball milling and hot pressing. The arc erosion behavior of the material was evaluated using homemade equipment. The microstructure and phase evolution of the materials were investigated through X-ray diffraction, energy-dispersive spectroscopy and scanning electron microscopy. The results showed that, although the mass loss of the Ag-SnO_2_-ZnO composite (9.08 mg) during the electrical contact test was higher than that of the commercial Ag-CdO (1.42 mg), its electrical conductivity remained constant (26.9 ± 1.5% IACS). This fact would be related to the reaction of Zn_2_SnO_4_’s formation on the material’s surface via electric arc. This reaction would play an important role in controlling the surface segregation and subsequent loss of electrical conductivity of this type of composite, thus enabling the development of a new electrical contact material to replace the non-environmentally friendly Ag-CdO composite.

## 1. Introduction

Ag-CdO composites (10.0–15.0 wt. % CdO), have been the preferred material for electrical contacts applications for many years [1] thanks to their high electrical and thermal conductivities [2,3], high resistance to arcing [3,4,5], high welding adhesion resistance [6,7], low contact resistance [6], hardness and strength [2,3]. Nevertheless, given the toxic nature of Cd and its compounds [7,8,9], the use of this element is expected to be minimized shortly. In this context, Directive 2011/65/EU of the European Parliament (RoHS 2011/65/EU) has restricted the use of Cd in electrical and electronic equipment. However, its use in electrical contact components has not been eliminated because no viable substitute for Ag-CdO composites has yet been developed.

For several decades, Ag-SnO_2_ materials (2.0–14.0 wt. % SnO_2_) have emerged as an environmentally friendly substitute for Ag-CdO [10]. Nevertheless, the application of Ag-SnO_2_ materials is still limited by two principal factors:

High contact resistance: Ag-SnO_2_ materials exhibit greater contact resistance than Ag-CdO composites [11,12]. This behavior is attributed to the formation of a SnO_2_-rich layer on the contact surface during device operation, due to the greater thermal stability of SnO_2_ than that of CdO, and the poor wettability between SnO_2_ and molten Ag [13,14]. 

Efforts have been made to improve the interfacial wettability between Ag and SnO_2_ by focusing on the addition of a second oxide. In this context, Wang et al. [15] reported that the incorporation of La_2_O_3_ improves the wetting ability of Ag-SnO_2_ composites, increasing the arc erosion resistance of the material. Recently, Cao et al. [16] studied the effect of La_2_Sn_2_O_7_ on the arc erosion behavior of Ag-SnO_2_ composites. They found that the addition of La_2_Sn_2_O_7_ reduces the contact angle and improves the wettability between molten Ag and SnO_2_, reducing the liquid Ag’s splashing. Nevertheless, the introduction of non-conductive oxides generally adversely affects the electrical conductivity of Ag-SnO_2_ composites [17].

Manufacturing problems: During the internal oxidation of Ag-Sn alloys, a surface oxidized film is formed, which prevents further progression of the internal oxidation by restricting oxygen diffusion through the superficial oxide [18,19]. As a result, the internal oxidation of Sn is not completed, especially when the Sn content is greater than approximately 5 wt. % of the Ag matrix [20,21].

The use of additional solute elements has been proposed to solve this problem. For example, Schimmel et al. [22] investigated the effect of In on the internal oxidation of Ag-Sn-In alloys. Their results showed that In_2_O_3_ promotes the heterogeneous nucleation of SnO_2_, thereby improving the internal oxidation of the alloy. Despite the improvements of the Ag-Sn internal oxidation process, this synthesis route produces an inhomogeneous microstructure formed by acicular SnO_2_ preferentially located at Ag grain boundaries [23]. This tends to make the contact behavior of the material inconsistent, because it is eroded [24]. Thus, Ag-SnO_2_ materials produced by internal oxidation cannot attain the high-grade functional properties of Ag-CdO composites. Today, powder metallurgy is the preferred route of Ag-SnO_2_ synthesis. Gavriliu et al. [25], Li et al. [26], and Wang et al. [27], among other authors, have studied the manufacturing of Ag-SnO_2_ composites by classical powder metallurgy techniques from very fine Ag-SnO_2_ powders obtained via chemical coprecipitation methods. Other powder metallurgy techniques have been employed for Ag-SnO_2_ synthesis. Wang et al. [28] reported the production of Ag-SnO_2_ composite by hot pressing, while Jiang et al. [29] studied the fabrication of this material by hot extrusion. Recently, the use of an electroless coating has been proposed to improve the interfacial relations between Ag and SnO_2_ [30,31]. The electrical material produced using these approaches has presented a uniform and fine SnO_2_ distribution in the Ag matrix. Alternatively, Wang et al. [13], Liu et al. [32] and Zhang et al. [33] have used a milling process to improve the homogeneity of the initial powders. However, although the formation of a dense SnO_2_ film can be avoided, the problems of poor wettability between SnO_2_ and Ag and the loss of conductivity due to the addition of dopants persist.

Zn_2_SnO_4_ has attracted extensive attention due to its good electrical conductivity [34] and high thermal stability [35]. Huai et al. [36] studied the production of Zn_2_SnO_4_ by a solid-state reaction between ZnO and SnO_2_. They found that these oxides begin to react at approximately 800 °C. In addition, Zn_2_SnO_4_ generally adheres well to an Ag matrix [37]. Notably, the effect of Zn_2_SnO_4_’s addition on the electrical contact properties of Ag has not yet been investigated.

Based on the higher thermodynamic stability of Zn_2_SnO_4_ in comparison with SnO_2_ and ZnO at high temperatures [35], SnO_2_ and ZnO are expected to react to form Zn_2_SnO_4_ on the contact surface during the regular electrical contact operation of an Ag-SnO_2_-ZnO composite. Thus, the oxide segregation on the surface of the Ag-based contact material during its regular operation could be reduced without using dopants, thereby solving one of the main problems presented by the Ag-SnO_2_ electrical contact materials.

In regard to the aforementioned, the main objective of this work was to study the synthesis of Ag-SnO_2_-ZnO composites by powder metallurgy methods and their subsequent arc erosion resistance in order to verify the superficial generation of Zn_2_SnO_4_ during their use as electrical contact materials.

## 2. Materials and Methods

### 2.1. Production of Ag-SnO_2_-ZnO Composite Powders

Powders of Ag (99.9 wt. % purity, particle size < 10 µm, Sigma-Aldrich, St. Louis, MO, USA), SnO_2_ (99.9 wt. % purity, particle size < 44 µm, Sigma-Aldrich), and ZnO (99.9 wt. % purity, particle size < 5 µm, Sigma-Aldrich) were mixed to compositions corresponding to Ag—11 wt. % SnO_2_—4 wt. % ZnO (composition I), Ag—7.5 wt. % SnO_2_—7.5 wt. % ZnO (composition II), and Ag—4 wt. % SnO_2_—11 wt. % ZnO (composition III). The compositions of the powders were established considering the typical relations between oxide phases and silver used in electrical contact materials [10]. The combinations of powders were ball-milled in Retsch E-max equipment (Retsch GmbH, Haan, Germany) to obtain a homogeneous and fine dispersion of oxide particles into the Ag matrix. All the millings were carried out under Ar protective atmosphere (99.95 wt. % purity, Linde, Woking, UK) using a stainless steel vessel (125 mL) and a ball-to-powder-weight ratio of 20 to 1. The millings were performed at 1500 rpm for 0.5 h and 2 h. Moreover, 1 wt. % stearic acid was added as a processing control agent to prevent excessive welding.

### 2.2. Production of Ag-SnO_2_-ZnO Composite Samples

Ag-SnO_2_-ZnO pieces were produced by the powder metallurgy route. To prevent swelling of the material during the sintering, the milled powders were outgassed by heating up to 400 °C for 2 h under a vacuum [38]. The powders were hot pressed in homemade equipment using a graphite die, with an inner diameter of 9.5 mm, at 600 °C under an Ar flow of 1.3 L min^−1^ for 3 h while applying a constant uniaxial pressure of 40 MPa. The thermal cycle comprised two stages. First, powders were heated to about 150 °C and held at that temperature for 10 min to remove volatile compounds. Second, the temperature was increased to the established working temperature, using a heating rate of 20 °C min^−1^. The pressure was maintained until complete cooling of the system to avoid pore formation. After the sintering, the pieces were repressed under 800 MPa for 10 min. All the sintering tests were duplicated to ensure the reproducibility of the results.

### 2.3. Characterization

The microstructural characteristics of the milled powders and sintered samples were studied by combining X-ray diffraction (XRD) and scanning electron microscopy (SEM). XRD analyses were carried out in a Shimadzu XRD-6000 diffractometer, Kyoto, Japan (Cu Kα radiation) using an angular step of 0.02° (2θ) and a counting time per step of 4 s. XRD patterns were analyzed by the Rietveld method [39], using the Material Analysis Using Diffraction (MAUD) program [40]. The morphology of the milled powders and the chemical homogeneity of some cross-sectioned samples were evaluated using a Zeiss model EVO MA10 (Oberkochen, Germany) thermionic microscopy equipped with an energy-dispersive X-ray analyzer Oxford model X-maxN 20 SDD. Some samples were analyzed in a Zeiss model Sigma 500 VP field emission gun SEM (FEG-SEM) in order to obtain high-resolution images. The particle size distribution of the milled powder was determined by laser diffraction, using Malvern model Mastersizer 2000 equipment (Malvern Panalytical Ltd., Malvern, UK).

The densities of the sintered samples were obtained through the Archimedes method according to ASTM B962. At the same time, the samples’ Vickers micro-hardness was measured in a Zwick Roell ZHVl-M machine (Ulm, Germany) using a load of 1000 g under ASTM E384. The electrical conductivity of the consolidated samples was also determined using a Sigma Scope SMP10 conductivity tester, according to ASTM E1004. 

### 2.4. Electrical Contact Test

**Figure 1:**
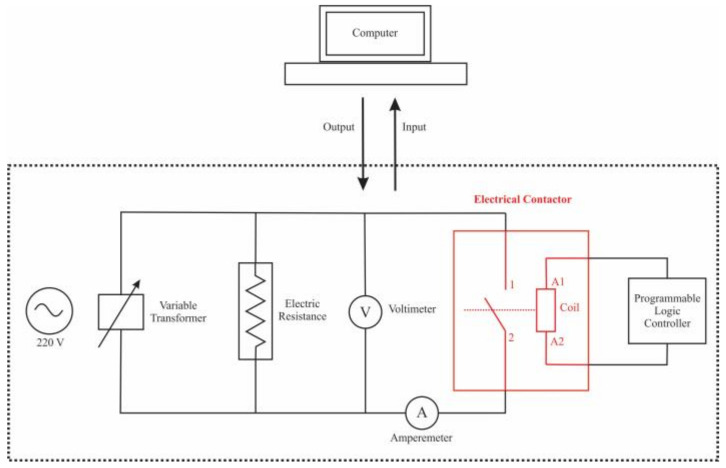
Schematic diagram of electrical contact systems.

The electrical contact tests of the Ag-SnO_2_-ZnO samples were carried out with homemade equipment. A schematic diagram of the system is shown in Figure 1. The sintered samples were installed in a commercial contactor Mitsubishi model S-N35 and tested for 5000, 10,000, 15,000, 20,000, and 25,000 on-off switching operations in a resistive circuit at a voltage of 220 V AC, current of 35 A and operation frequency of 30 times per minute. The mass loss of the samples was measured in a precision electronic scale Denver Instrument model TP-124. The morphology and chemical composition of the eroded contact surface were analyzed by FEG-SEM. The variation of electrical conductivity during the contact tests was determined using the Sigma Scope SMP10 equipment (London, UK). Additionally, the microstructure present in the eroded surface was analyzed by XRD.

## 3. Results

### 3.1. Morphological and Microstructural Examination of the Powders

**Figure 2:**
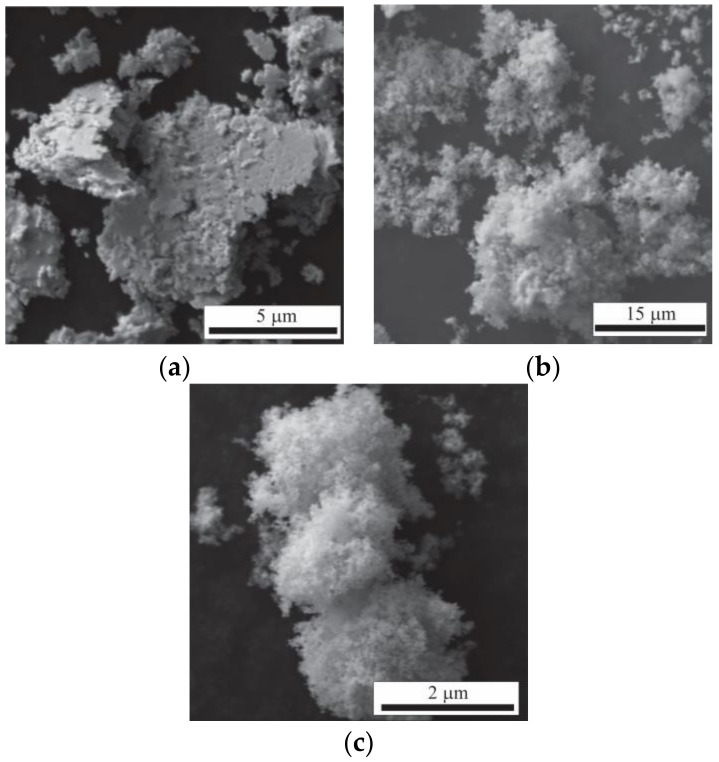
Morphology of initial powders. (**a**) Ag, (**b**) SnO_2_ and (**c**) ZnO.

The morphology of the starting powder is shown in Figure 2. It can be seen that the Ag powders are formed by flake-shape agglomerates, while the oxide powders (SnO_2_ and ZnO) are constituted by rounded agglomerates from fine particles. The maximum agglomerate size observed is in accordance with that reported by the manufacturer in each case (Ag < 10 µm, SnO_2_ < 44 µm, and ZnO < 5 µm).

**Figure 3:**
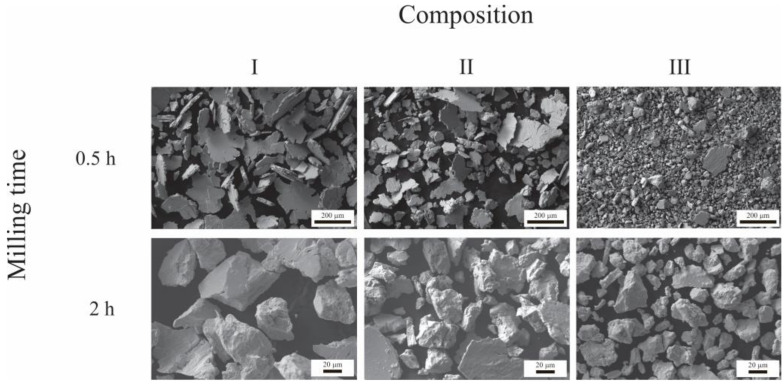
Morphology of the powders after 0.5 h and 2 h of milling for the three compositions under study.

Figure 3 shows SEM images of the powders milled for 0.5 h and 2 h for the three compositions under study. It can be seen that, after 0.5 h of milling, the powders comprise flat agglomerates produced due to the ductile nature of Ag, which is plastically deformed. After 2 h of milling, the morphology of certain agglomerates changes from lamellar to equiaxed. This occurs due to an increase in the density of dislocations and the powders’ embrittlement, causing the fracture to prevail over plastic deformation [41].

**Figure 4:**
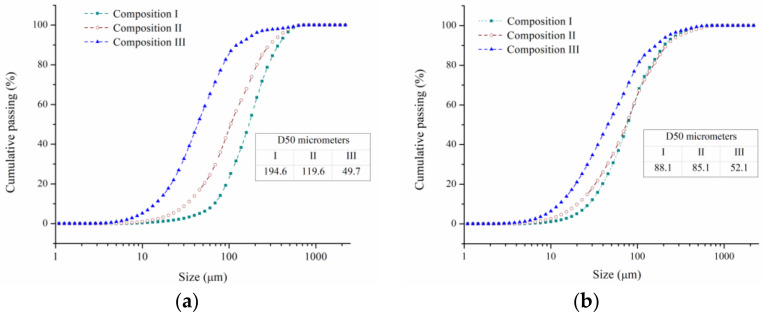
Particle size distributions of the powders after (**a**) 0.5 h and (**b**) 2 h of milling for the three compositions under study.

Additionally, the particle size distribution decreased with increasing ZnO content in the initial mixture. This can be seen more clearly in Figure 4, which shows the particle size distributions of the powders after 0.5 h and 2 h of milling for the three compositions. This behavior can be explained by considering the effect of the initial particle size of oxides (SnO_2_ and ZnO) on the milling process. Since ZnO (<5 µm) has a smaller particle size than SnO_2_ (<44 µm), ZnO particles act as a more effective obstacle against the dislocation movement than SnO_2_ during milling. It is well known that the fine non-shearing ceramic particles pin the crossing dislocations and promote dislocation bowing around the particles under external load (Orowan loops). This fact reduces the toughness of the powders and promotes their fragmentation during the milling process. This behavior is accentuated with the decrease in the particle size of the ceramic phase, since the interparticle distance between the dispersoids decreases [42].

In this sense, the powders of composition III (higher ZnO content) seem to have reached their comminution limit because no significant change in the particle size distribution was observed between 0.5 h and 2 h of milling. This behavior suggests that a balance between fracturing and cold welding has been achieved for this composition under the experimental conditions employed. 

**Figure 5:**
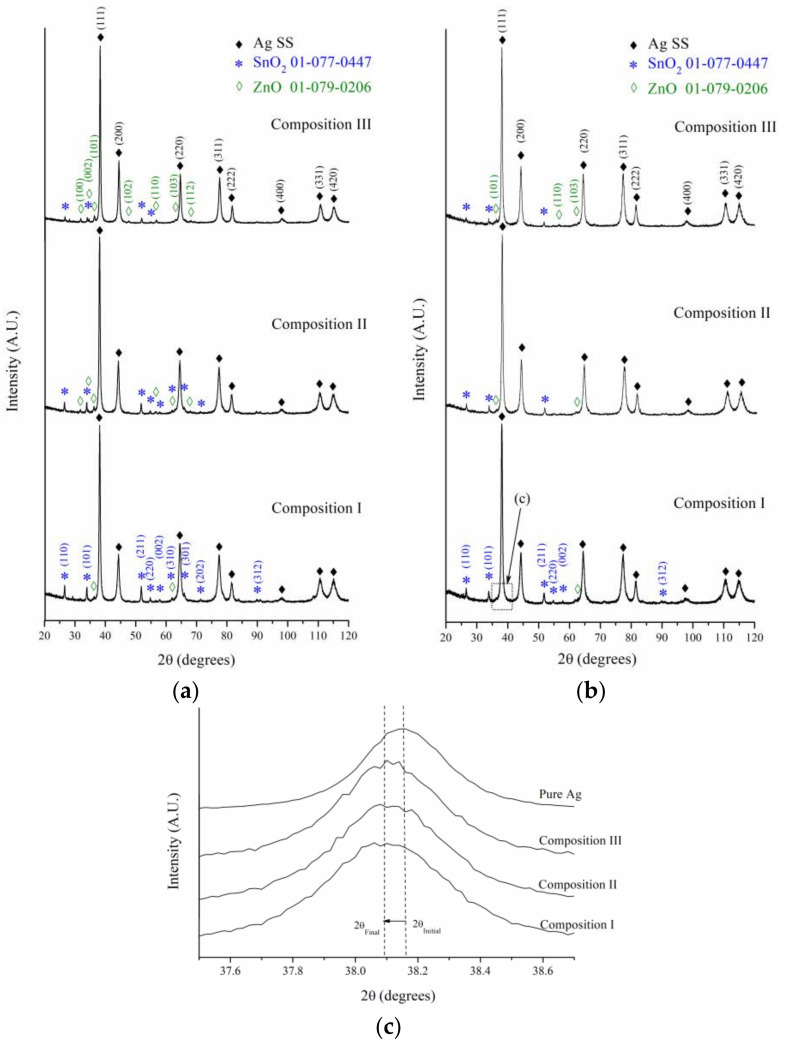
XRD patterns of powders milled for (**a**) 0.5 h and (**b**) 2 h. (**c**) Magnified zone of the selected area shown in (**b**).

Figure 5 shows the XRD patterns of the powders after 0.5 h and 2 h of milling. For a comparison between the XRD patterns, they were normalized in regard to the maximum intensity. After 2 h of milling, the relative diffraction intensity of ZnO decreased at a greater proportion than that of the SnO_2_. This behavior is due to the fact that ZnO is more than six times softer than SnO_2_ (the microhardness of ZnO and SnO_2_ are 1.5 GPa and 10 GPa, respectively). As a result, the size reduction of ZnO was accelerated during the milling process [43]. Besides, the formation of a new oxide product of the mechanical reaction between ZnO and SnO_2_ was not detected.

**Figure 6:**
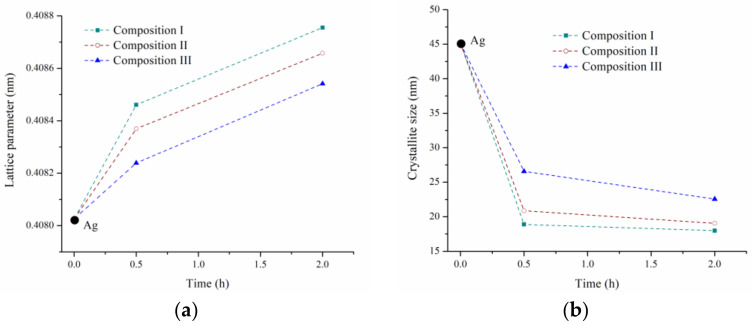
(**a**) Lattice parameter and (**b**) crystallite size of Ag-rich solid solution as a function of milling time and composition.

Figure 5c presents a magnified zone for the diffraction of the (111) plane of Ag for the samples milled during 2 h. The XRD pattern of pure Ag was incorporated for comparison purposes. A shift was observed in the position of the diffraction peaks of the milled powders toward lower 2θ angles. This behavior is related to the unit cell expansion of Ag due to the formation of a solid solution during the milling process. In this sense, Figure 6a shows the variation of the lattice parameter of Ag-rich solid solutions (Ag SS) as a function of milling time and composition. The lattice parameters were obtained from Rietveld refinements, using the MAUD software [40]. The lattice parameter of the solid solution increased with increasing SnO_2_ content in the initial combination of powders. It has been reported that, unlike Zn, whose incorporation into the Ag causes a contraction of its unit cell [44], Sn’s incorporation generates an expansion in the crystal structure of Ag due to its larger atomic volume, Ω (Ω_Sn_ 0.054 nm^3^ atom^−1^, Ω_Ag_ 0.017 nm^3^ atom^−1^) [45]. In this sense, the results obtained, supported by the evidence in the literature, suggest that, during the milling process, part of the SnO_2_ was destabilized, leading the Sn into the crystalline structure of the Ag matrix, thus forming a solid solution whose Sn concentration increases with increasing SnO_2_ content in the initial powders. At this point, the incorporation of small amounts of Zn into Ag during the milling process could not be ruled out. Further discussion of this topic will be presented in the following sections.

Figure 6b shows the variation of the crystallite size of the Ag-rich solid solution with regard to the milling time and composition. This was calculated from the XRD patterns via Rietveld refinement in the MAUD program [40]. The crystallite size of the initial pure Ag was incorporated for comparison purposes. The crystallite size of the Ag solid solution rapidly reduced during the first 0.5 h of milling due to the high plastic deformation to which the powders were subjected during the milling process. Afterwards, the crystallite size seemed to have reached a minimum value. This steady state is determined by the balance between the processes that tended to decrease the crystallite size (plastic deformation and dislocation motion) and those that increased the size (recovery and recrystallization) [46]. 

Additionally, there was an inverse relationship between the crystallite size of the Ag solid solution and the SnO_2_ content in the initial combination of powders. This behavior could be explained by the higher Sn content present in the Ag-rich solid solution generated from the powders with a higher initial concentration of SnO_2_ (Composition I > Composition II > Composition III). The highest Sn content increases the activation energy for the recovery and recrystallization of the Ag solid solution [47], making it difficult to verify these processes during the milling, promoting a smaller final size of crystallite due to the increase in the density of dislocations and crystalline defects as a result of the severe plastic deformation.

**Figure 7:**
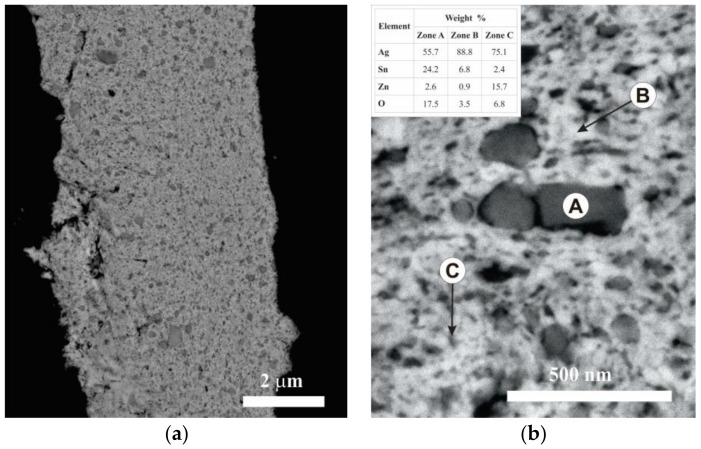
(**a**) BSE image of cross-sectioned powders of composition II milled for 2 h and (**b**) magnified view. The table presents the average of three EDS analyses of selected zones.

To determine the distribution of the oxides, powders milled for 2 h were metallographically prepared and observed by FEG-SEM, using backscattered electrons (BSE). For example, Figure 7 shows the results obtained from the powders of composition II. Two particle size distributions were identified. The first is characterized by particles with a large relative size (equivalent diameter between 50 and 300 nm). The EDS analyses of these regions indicated a high presence of Sn and O (Zone A in Figure 7b), suggesting that these particles would correspond principally to SnO_2_. The second distribution is formed by very fine particles (equivalent diameter < 30 nm). The EDS analyses of these sectors showed a high concentration of Zn and O (Zone C in Figure 7b), indicating that they would correspond principally to ZnO. Similar results were found in the three compositions under study. 

The difference in size between the particles of SnO_2_ and ZnO after the milling process can be attributed to two factors: the lower hardness of ZnO (1.5 GPa) concerning SnO_2_ (10 GPa), which accelerates its size reduction during the milling process, and the difference in size between the initial powders of SnO_2_ (particle size < 44 µm) and ZnO (particle size < 5 µm). 

Finally, the EDS analyses carried out on the matrix (Zone B in Figure 7b) indicated that it presents a higher concentration of Sn than Zn. These results confirmed that Sn preferentially solubilized in the Ag matrix during the milling process. This confirmed the XRD results.

### 3.2. Physical and Microstructural Characterization of the Sintered Samples

**Figure 8:**
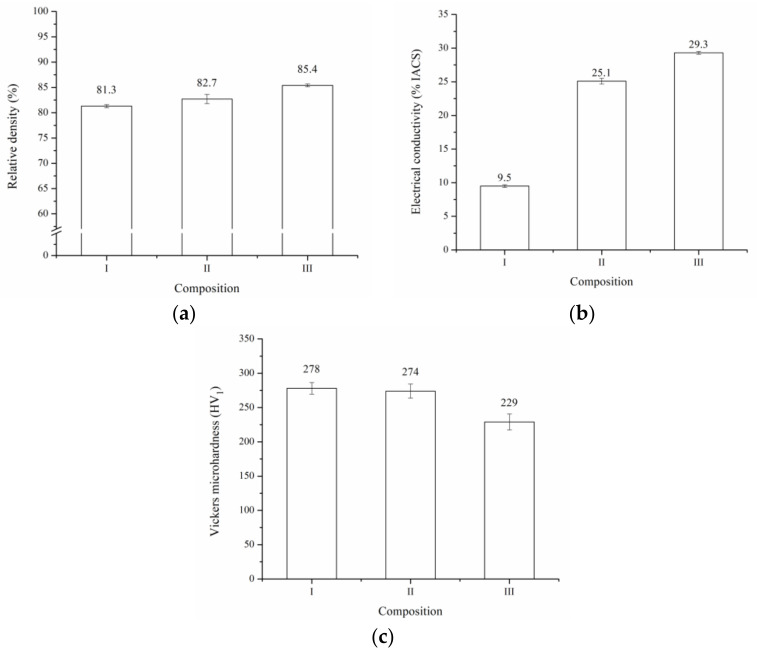
(**a**) Relative density (**b**) electrical conductivity and (**c**) Vickers microhardness for the three compositions under study.

The powders were hot pressed at 600 °C for 3 h under a constant uniaxial pressure of 40 MPa. Subsequently, the pieces were repressed under 800 MPa for 10 min. Figure 8a shows the relative density for the three compositions under study. The relative density increased with increasing ZnO content in the initial combination of powders. This behavior could be explained by the smaller particle size that reached the powders of composition III during the milling process (Figure 4b). In this sense, it is well known that the decrease in particle size promotes densification during the sintering process due to the increase in the surface energy of the system [48]. 

**Figure 9:**
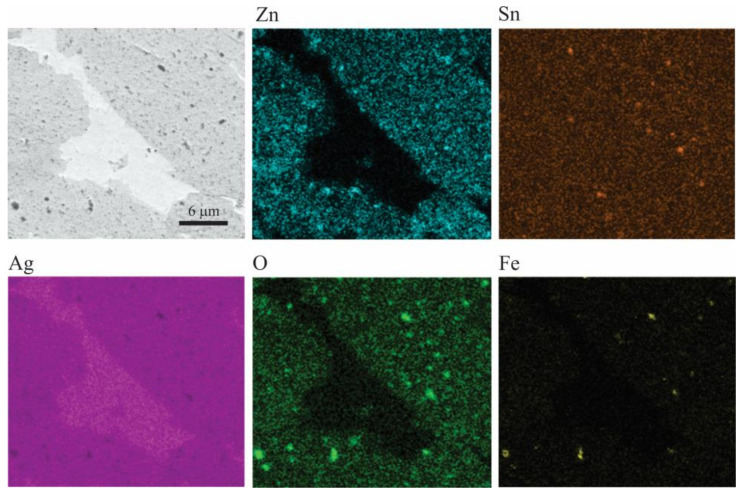
BSE image of a cross-sectioned sintered sample of composition II and EDS elemental mapping.

The relation between the electrical conductivity and composition is shown in Figure 8b. The electrical conductivity of the samples increased with increasing ZnO content in the initial combination of powders. This is mainly due to the reduction of porosity as a result of the increase in the relative density (Figure 8a) and the reduction of the Sn content in the Ag-rich solid solution (Figure 6a). It has been reported that the electrical conductivity of Ag solid solutions decreases sharply with the increase of solute content [49]. The above becomes relevant if we consider that, during the sintering process, a preferential diffusion of the Ag-Sn solid solution occurs, producing regions that are free of oxides (SnO_2_ and ZnO) and Zn, as seen in Figure 9, which shows as an example a BSE cross-sectional image of a sintered sample of composition II and its respective EDS elemental mapping. This analysis also detected small Fe-rich areas, which indicates contamination from the erosion of balls and vial during the milling process. 

Finally, Figure 8c shows the Vickers microhardness for the three compositions. The sample with the composition I (higher SnO_2_ content) exhibited a higher microhardness than its counterparts. The above could be attributed principally to the higher hardness of SnO_2_ compared with ZnO and the higher Sn content of the Ag-rich solid solution in this sample (Figure 6a). In this sense, it has been reported that the hardness of the dispersed particles plays a relevant role in their ability to inhibit the dislocation movement in a metallic matrix [50].

When the results obtained were analyzed, it could be established that the microhardness reached by Ag-ZnO-SnO_2_ composites was higher than that of the commercial Ag-CdO/Ag-SnO_2_/Ag-ZnO materials (<110 HV) [51,52,53,54]. This would be mainly due to the fine and homogeneous distribution of oxides produced during the milling process (Figure 7). However, this fine distribution of oxide negatively affected the electrical conductivity of this type of material, which was below that reported for commercial composites (>65% IACS). Therefore, it is recommended that future studies optimize the size and distribution of the oxide particles and the variables of the sintering process to achieve a higher degree of densification, in order to improve the electrical conductivity of the Ag-ZnO-SnO_2_ composites. Additionally, to reduce the production cost of this material, the use of powders composed of Cu-ZnO-SnO_2_ electroless coated with a layer of Ag could be tested [55].

### 3.3. Arc Erosion Behavior of Ag-SnO_2_-ZnO

**Figure 10:**
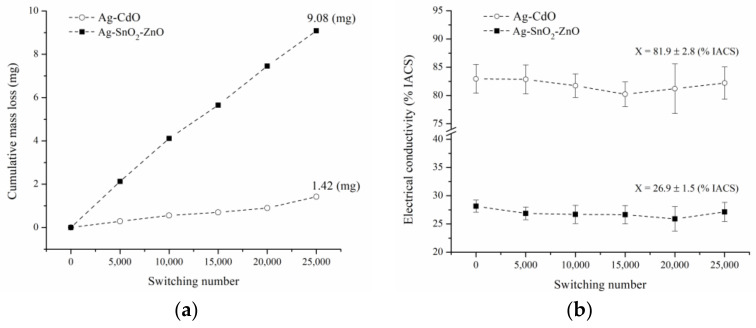
(**a**) Cumulative mass loss and (**b**) electrical conductivity as a function of switching number.

Due to their higher densification and electrical conductivity, sintered samples of composition III were selected to be tested for 25,000 on-off switching operations in a resistive circuit at 220 V AC and 35 A. Composites samples of composition I and II were not tested. A commercial Ag-CdO contact material was submitted to the same test conditions for comparison purposes. The composite Ag-SnO_2_-ZnO presented a more significant mass loss than the commercial Ag-CdO (Figure 10a), which is mainly due to the low densification reached during the sintering process. It is well known that porosity negatively affects the arc erosion resistance of the materials [56]. Figure 10b shows the electrical conductivity as a function of the on-off switching operations. As seen, there was no appreciable variation in the electrical conductivity for both materials during the electrical contact tests.

**Figure 11:**
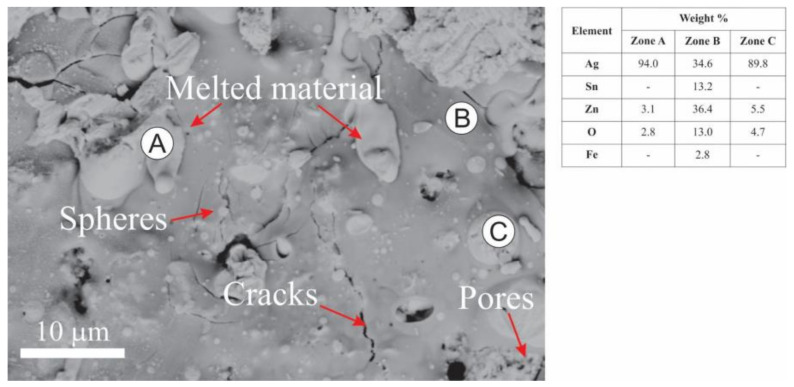
BSE image of the surface of the Ag-SnO_2_-ZnO contact material after arc erosion and EDS analyses of selected areas.

Figure 11 shows the surface morphology of eroded material and EDS analyses of marked regions. The action of the electric arc generated the segregation of the elements on the material surface. The high energy dissipated by the electric arc melted the surface layers of the contact, generating regions rich in Ag (Zone A) and sectors with a high concentration of Zn, Sn, and O (Zone B). The appearance of these segregated areas could be explained by the poor wettability between the oxides (ZnO/SnO_2_) and molten Ag [57]. Additionally, small spheres with diameters ranging from several hundred nanometers to ~10 µm can be observed on the surface, which presented a high Ag concentration. Those spheres were principally formed by the splash of liquid Ag during arc erosion. 

Tiny pores and several cracks were observed on the eroded surface. The pores were produced mainly due to gases escaping from the melted Ag-rich solution products of its rapid solidification [57]. At the same time, the cracks were generated because of the accumulation of stresses during the cooling due to the different thermal expansion coefficients between Ag and the oxides (SnO_2_ and ZnO) [58].

**Figure 12:**
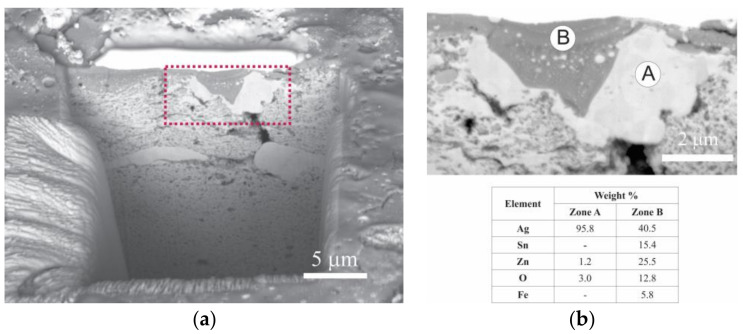
(**a**) BSE image of cross-sectioned sampled using FIB (**b**) magnified image and EDS analyses of selected areas.

For an in-depth study of the eroded surface, the piece was cross-sectioned with a FIB and analyzed by FEG-SEM. Figure 12 shows the results obtained. A surface layer with a high concentration of Sn, Zn and O generated due to the action of the electrical arc (Zone B) can be observed. Under this layer, a region with a high Ag concentration is localized (Zone A). Moreover, it is possible to detect tiny particles of the Ag-rich phase dispersed within the oxide surface layer. As mentioned above, this stratification phenomenon is related to the poor wettability between molted Ag and the oxides (SnO_2_ and ZnO), which produces segregation on the material’s surface [57].

**Figure 13:**
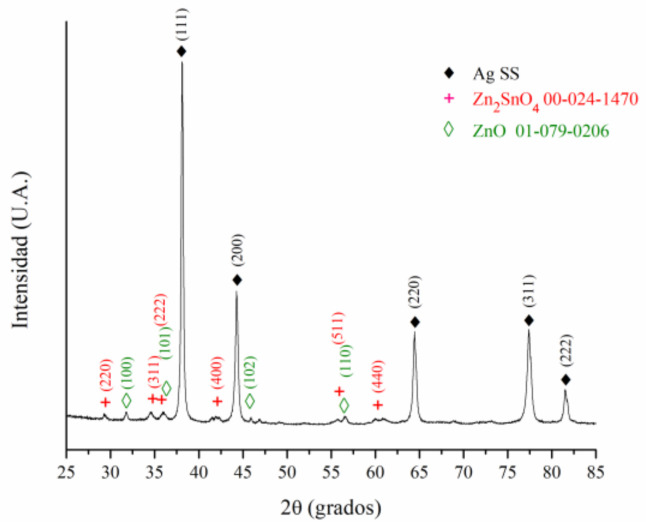
XRD patterns of arc eroded sample.

Based on the BSE images of the cross-sectioned sample, it could be concluded that the surface layer of the oxide presents a very homogeneous chemical composition. This could be attributed to a possible reaction between SnO_2_ and ZnO during the electrical contact test. To verify this supposition, the eroded sample was analyzed by XRD. The results obtained can be seen in Figure 13. The presence of an Ag solid solution, ZnO and Zn_2_SnO_4_ was detected, confirming the reaction between SnO_2_ and ZnO on the surface of the material according to the following reaction:

SnO_2_ + 2 ZnO → Zn_2_SnO_4_ (1)

**Figure 14:**
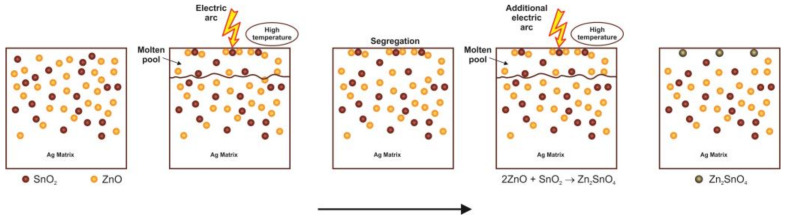
Schematic of the performance of the Ag-SnO_2_-ZnO electrical contact material.

To explain the results obtained, we proposed the following reaction mechanism, schematically presented in Figure 14. In the initial stage, a superficial layer of the material is melted due to arc energy, forming a molten pool where the SnO_2_ and ZnO are segregated on the surface due to their lower density and poor wettability with liquid Ag. In the intermediate stage, the segregation process continues, promoting the contact between the particles of SnO_2_ and ZnO. In the last stage, the reaction between SnO_2_ and ZnO to form Zn_2_SnO_4_ takes place due to the increase in temperature caused by the effect of electric arcs. 

Although the mass loss of the Ag-SnO_2_-ZnO composites during the electrical contact test was higher than that of the commercial Ag-CdO, their electrical conductivity remained constant. This fact would be related to the reaction of the formation of Zn_2_SnO_4_ on the surface of the material by the action of the electric arc (reaction (1)). Since the Zn_2_SnO_4_ has excellent adhesion to the Ag [37], the formation of this oxide would contribute to limit the superficial segregation, stabilizing sectors rich in Ag within the oxide surface layer (Figure 12b). Additionally, the formation reaction of Zn_2_SnO_4_ involves a volume contraction of 4%, which reduces the total amount of oxides in the superficial layer. This work could mark the starting point for developing a new strategy to limit the surface segregation of the Ag-SnO_2_/ZnO electrical contact materials, based on a superficial reaction of their components promoted by the electrical arc action during its regular operation.

## 4. Conclusions 

In this work, the synthesis of Ag-SnO_2_-ZnO composite by powder metallurgy methods and its subsequent electrical contact behavior were studied. The following main conclusions were obtained.

With regard to the morphology evolution of the powders during the milling process, the particle size distribution decreased with increasing ZnO content in the initial combination of powders. In the early milling stage, flat agglomerates were observed due to the ductile nature of Ag. As the milling time increased, the powders were welded and fractured until they reached an equiaxed morphology. Based on the XRD and SEM-EDS analyses, it could be established that the milling process promoted the formation of a fine and homogeneous distribution of SnO_2_ and ZnO in an Ag-Sn solid solution matrix.

Regarding the production of Ag-SnO_2_-ZnO pieces by hot pressing, the relative density and electrical conductivity increased with increasing ZnO content in the initial combination of powders, while the microhardness decreased. The pieces obtained presented a low electrical conductivity (21.3% IACS on average) compared with that of the commercial composites (>65% IACS. This would be mainly due to the fine and homogeneous distribution of oxides produced during the milling process and the insufficient densification reached in the sintering stage (83.1% on average).

Concerning the arc erosion behavior of the material, although the mass loss of the Ag-SnO_2_-ZnO composites during the electrical contact test was higher than that of the commercial Ag-CdO, its electrical conductivity remained constant. This fact would be related to the reaction of the formation of Zn_2_SnO_4_ on the material’s surface by the action of the electric arc. This reaction would play an important role in controlling the surface segregation and subsequent loss of electrical conductivity of this type of material.

Finally, this study could lead to the development of a new electrical contact material to replace the non-environmentally friendly Ag-CdO composites used in low-voltage applications. In this sense, it is recommended that future works optimize the size and distribution of the oxide particles and the variables of the sintering process to achieve a higher degree of densification to improve the electrical contact performance of this new material.

## Data Availability

Not applicable.
